# The Development of a Repetition-Load Scheme for the Eccentric-Only Bench Press Exercise

**DOI:** 10.2478/hukin-2013-0042

**Published:** 2013-10-08

**Authors:** Gavin L. Moir, Kyle F. Erny, Shala E. Davis, John J. Guers, Chad A. Witmer

**Affiliations:** 1 Exercise Science Department, East Stroudsburg University, East Stroudsburg, PA, USA.

**Keywords:** bench press, eccentric contraction, repetition-load scheme

## Abstract

The purpose of the present study was to develop a repetition-load scheme for the eccentric-only bench press exercise. Nine resistance trained men (age: 21.6 ± 1.0 years; 1-repetition maximum [RM] bench press: 137.7 ± 30.4 kg) attended four testing sessions during a four week period. During the first session each subject’s 1-RM bench press load utilizing the stretch-shortening cycle was determined. During the remaining sessions they performed eccentric-only repetitions to failure using supra-maximal loads equivalent to 110%, 120% and 130% of their 1-RM value with a constant cadence (30 reps·min^−1^). Force plates and a three dimensional motion analysis system were used during these final three sessions in order to evaluate kinematic and kinetic variables. More repetitions were completed during the 110% 1-RM condition compared to the 130% 1-RM condition (p=0.01). Mean total work (p=0.046) as well as vertical force (p=0.049), vertical work (p=0.017), and vertical power output (p=0.05) were significantly greater during the 130% 1-RM condition compared to the 110% 1-RM condition. A linear function was fitted to the number of repetitions completed under each load condition that allowed the determination of the maximum number of repetitions that could be completed under other supra-maximal loads. This linear function predicted an eccentric-only 1-RM in the bench press with a load equivalent to 164.8% 1-RM, producing a load of 227.0 ± 50.0 kg. The repetition-load scheme presented here should provide a starting point for researchers to investigate the kinematic, kinetic and metabolic responses to eccentric-only bench press workouts.

## Introduction

It has been demonstrated during in vitro and in vivo skeletal muscle actions that the tension developed under eccentric conditions exceeds that developed during a concentric contraction (Dudley et al., 1990; Harry et al., 1990; Worrell et al., 1991). Due to the greater muscular tension developed, eccentric muscle contractions have been proposed to provide a more potent stimulus for both hypertrophy and strength gains following a period of resistance training (Roig et al., 2009; Schoenfeld, 2010). Most of the current research has compared the effects of eccentric contractions to concentric contractions performed using isokinetic dynamometers, devices that few practitioners have access to, during lower-body, single-joint exercises. There is little information on the effects of eccentric contractions on multi-joint, upper-body exercises. Furthermore, few studies have employed supra-maximal loads; that is, loads greater than those achieved in either maximal concentric-only contractions or those achieved during exercises utilizing the stretch-shortening cycle (SSC - combining eccentric and concentric contractions i.e. typical 1-repetition maximum [1-RM] tests).

Crewther et al. (2005) noted that although the training load used during resistance training exercises often determines the specific adaptations accrued, the kinematics and kinetics associated with the movement of the load may be more important. Furthermore, these authors noted that currently very little is known about the kinematics and kinetics associated with resistance training movements utilizing eccentric contractions performed under supra-maximal loading conditions. This is an important omission from the extant literature as it limits the practitioners’ ability to effectively prescribe effective supra-maximal loads. However, the issue is compounded by the lack of repetition-load schemes for eccentric-only resistance training movements unlike the schemes that have been developed for concentric and stretch-shortening cycle movements (Baechle et al., 2008). Therefore, the purpose of the present study was to develop the repetition-load scheme during supra-maximal, eccentric-only bench press. Furthermore, the kinematic and kinetic differences between eccentric-only repetitions of the bench press performed to failure using supra-maximal loads equivalent to 110%, 120% and 130% of those derived from a typical 1-RM test (i.e. a 1-RM test utilizing the SSC) were investigated. This data could be used to design resistance training workouts to develop strength and hypertrophy using supra-maximal loads in the bench press exercise.

## Material and Methods

In order to investigate the changes in mechanical variables during eccentric-only bench press repetitions performed to failure with supra-maximal loads, resistance-trained men attended four testing sessions during a four week period. During the first session each subject’s 1-RM bench press load was determined. During the remaining sessions they performed eccentric-only repetitions to failure using supra-maximal loads equivalent to 110%, 120% and 130% of their 1- RM value. Force plates and a three dimensional (3-D) motion analysis system were used during these final three sessions in order to calculate associated mechanical variables.

### Participants

Nine resistance trained men (age: 21.6 ± 1.0 years; mass: 90.7 ± 21.0 kg; height: 1.76 ± 0.10; 1- RM bench press: 137.7 ± 30.4 kg) volunteered to participate in this study which was approved by the Institutional Research Board of East Stroudsburg University. Each subject had a minimum of one year resistance training experience that included bench press exercises in their workouts. Each subject signed an informed consent form prior to any testing.

### Data Collection

Each subject attended four testing sessions across a four week period, the first of which was used to determine their 1-RM load for the bench press. During the remaining three sessions each subject performed eccentric-only bench press repetitions to failure using supra-maximal loads equivalent to 110%, 120% and 130% 1-RM. The order that the supra-maximal eccentric trials were performed was randomized across the subjects and a minimum of one week was allowed between testing sessions.

#### 1-RM bench press protocol

The 1-RM load achieved during the bench press exercise was determined using the protocol outlined by Baechle et al. (2008). The bench press technique required the subject to lower the barbell from a position with the elbows fully extended to just above the chest before raising it back to starting position, so incorporating the SSC. The exercise was performed using an Olympic barbell and plates within a power rack.

#### Supra-maximal eccentric bench press protocol

The technique used during each experimental session was that outlined by Earle and Baechle (2008) with spotters placed at either end of the barbell and one placed on either side of the subject lifting. Each repetition was started with the subject accepting the barbell from the spotters with the elbows extended. The barbell was then lowered by the subject to a position just above the chest, from where the spotters then returned it to the starting position. The subjects maintained their grip on the barbell during the ascent back to the starting position but were instructed not to contribute to the ascent. The same grip width was ensured for each load condition. Adjustable support bars were added to the power rack at a level just above each subject’s chest as a safety precaution.

When performing multiple repetitions to failure in a given exercise the cadence selected can have a significant impact on the number of repetitions completed (LaChance and Hortobaygi, 1994). A cadence of 30 reps·min^−1^ (a 2 second eccentric phase) was selected in the present investigation following the recommendations of Ratamess et al. (2009), with the cadence being set using an electronic metronome (Seiko, model SQ70) and the subjects were familiarized with the cadence at the beginning of each testing session. The time taken for the spotters to lift the barbell back to the starting position was approximately 0.30 seconds and the barbell was supported by the spotters prior to the initiation of the next eccentric contraction by the subject in order to maintain the cadence. During each repetition the subjects were verbally encouraged and were provided with feedback on their cadence. Failure was determined when the subject could no longer maintain the required cadence or they indicated that they could no longer continue. During data analysis it was noted that the subjects were unable to produce an exact cadence of 30 reps·min^−1^, and so it was determined that any repetitions that exceeded a cadence of 60 reps·min^−1^ (a 1 second eccentric phase) were to be excluded from the analysis. This cadence still falls within the recommendations in the literature (Ratamess et al., 2009). Furthermore, from the negative displacement of the barbell during the descent, it was established that if the barbell was falling under gravity alone with no resistance offered by the subject the descent would take approximately 0.4 seconds.

Prior to each supra-maximal loading condition, all subjects completed the same warm-up comprising dynamic activities for the upper-body (e.g. arm circles). Each subject then performed eccentric-only bench press repetitions with a load equivalent to 50% of their 1-RM value for 10 repetitions, followed by 6 repetitions with a load equivalent to 80% of their 1-RM value, with two minutes of rest between the sets.

#### Calculation of mechanical variables

The bench was placed on two force plates (Kistler Type 9286AA) during the supra-maximal eccentric bench press sessions, with one plate at the subjects’ head and the other at their feet and it was ensured that the subject’s feet remained on this force plate during each repetition. The force plates were synchronized with a 3-D motion analysis system (Vicon, Oxford, UK) that collected the position of a retro-reflective marker placed in the middle of the barbell. The force plates and the 3-D system sampled the data at a frequency of 200 Hz. The ground reaction force data from the force plates and the position data from the 3-D system were used to calculate the following mechanical variables:

#### Number of repetitions

The total number of repetitions completed before failure performed under each supra-maximal loading condition was recorded. A repetition was defined as the event when the vertical velocity of the retro-reflective marker was negative. The vertical velocity was calculated using the first central difference method.

#### Volume-load (VL)

The product of the load and the number of repetitions was used to calculate volume-load.

#### Time under tension (TUT)

The time between the beginning of the eccentric phase of each repetition, defined as the first instance of negative vertical velocity of the barbell marker, and the end of the eccentric phase, defined as the next instance of positive vertical velocity, was used to represent the time under tension.

#### Force (F)

The ground reaction forces in the *x* (mediolateral), *y* (anterioposterior), and *z* (vertical) directions recorded from the two force plates were summed and then averaged across each repetition. The average force was then normalized to body mass using the allometric parameter of ⅔ (Jaric, 2002) to provide normalized force.

#### Impulse

The average force in the *x*, *y*, and *z* directions during each repetition was multiplied by TUT to provide the vertical impulse. The impulse was then normalized to body mass⅔ (Jaric, 2002).

#### Work

The work performed during each repetition was calculated by integrating the product of instantaneous force and velocity (power output) in the *x*, *y*, and *z* directions using the trapezoid rule. Work was then normalized to body mass⅔ (Jaric, 2002) and the values were converted to positive for ease of interpretation. Work was reported as both the sum of the work performed in the *x*, *y*, and *z* directions during each repetition (Total work) as well as being separated into that performed in the *x*, *y*, and *z* directions (Work).

#### Power output (PO)

The force was multiplied by the velocity of the barbell during each repetition to provide power output in the *x*, *y*, and *z* directions. The instantaneous power output was then averaged during each repetition and then normalized to body mass⅔ (Jaric, 2002) to provide normalized power output. The PO values were converted to positive for ease of interpretation.

The mechanical variables were averaged across the number of repetitions completed during each loading condition to provide mean values (TUT_mean_, F_mean_, Impulse_mean_, Total work_mean_, Work_mean_, PO_mean_). Total work was also summed across all repetitions during each load condition to provide the cumulative total work (Total work_cum_).

### Statistical Analysis

All statistical analyses were performed using the Statistical Package for Social Sciences (SPSS version 18.0). Measures of central tendency and spread of the data were represented as means and standard deviations. Differences in the total number of repetitions completed and the mean mechanical variables recorded during each supra-maximal loading condition were assessed using an ANOVA model with repeated measures on one factor (load condition: 3 levels). Pairwise comparisons with Bonferoni corrections were used to determine where the differences occurred. The level of statistical significance for all analyses was set at p ≤ 0.05. The number of repetitions completed under each load condition was analyzed to determine the function that fitted the data in order to allow the determination of a repetition-load scheme for the eccentric-only bench press and the prediction of an eccentric 1-RM (1-RM_ECC_).

## Results

Table 1 shows the total number of repetitions performed and the volume-load during the supra-maximal loading conditions of 110%, 120% and 130% 1-RM as well as the mean values for the mechanical variables of TUT, Total workmean, and Total workcum.

There were significantly more repetitions performed with a load equivalent to 110% 1-RM compared to the 130% 1-RM condition (p = 0.01) while the Total workmean performed during each repetition was significantly greater with a load equivalent to 130% 1-RM compared to the 110% 1-RM condition (p = 0.046).

Table 2 shows the mean values for the mechanical variables in the x, y, and z directions recorded during the different load conditions.

The repetitions performed with a load equivalent to 130% 1-RM produced significantly greater Fmean in the z direction compared to the 110% 1-RM condition (p = 0.049). Furthermore, significantly greater Workmean (p = 0.017) and POmean (p = 0.050) in the z direction were produced in the 130% 1-RM condition compared to the repetitions performed with the 110% 1-RM load. There were no significant differences for any of the mechanical variables recorded in the x and y directions between the different load conditions.

A linear function was fitted to the number of repetitions performed under each loading condition.

This linear function had the following equation (R^2^ = 0.99):

y=−2.3889x+16.481

where, y = number of repetitions x = loading (i.e. loading 0 = 100% 1-RM; loading 1 = 110% 1-RM; loading 2 = 120% 1-RM etc.)

From this linear function a 1-RMECC was predicted with a load equivalent to 164.8% 1-RM, producing a 1-RMECC load of 227.0 ± 50.0 kg for the subjects. Table 3 shows the actual number of repetitions performed under the different loading conditions as well as the number of repetitions predicted under the different loading conditions calculated from the linear function.

The loads are also expressed as a percentage of 1-RMECC in this Table.

## Discussion

The first purpose of the present study was to develop an eccentric-only repetition-load scheme for the bench press. The repetition-load scheme was developed from the linear function applied to the repetitions to failure performed under the three supra-maximal loading conditions used in the present study. This linear function was able to accurately predict the number of repetitions achieved under the load conditions of 110, 120 and 130% 1-RM (Table 3). Furthermore, an eccentric-only 1-RM (1-RMECC) was calculated as 164.8% of 1-RM. There are no previous data available to determine the magnitude of the difference between a 1-RM performed in an eccentric-only bench press and a 1-RM performed during the exercise involving an eccentric phase (barbell descent) preceding a concentric phase (barbell ascent), and therefore the accuracy of the predicted 1-RMECC is difficult to ascertain. Hollander et al. (2007) reported that the 1-RMECC for the bench press was 140% of a concentric-only 1-RM bench press in a group of healthy young men. One would expect the load lifted during a movement involving the coupling of eccentric and concentric contractions to be greater than that lifted using concentric-only actions (Wilson et al., 1991), and so the value of 164.8% calculated in the present study may initially appear somewhat high. However, the cadence used by Hollander et al. (2007) was lower than that used in the present study (20 reps·min^−1^ versus ∼38 reps•min^−1^) which would imply greater eccentric forces were being generated by the subjects in the present study given the force-velocity relationship of skeletal muscle (Worrell et al., 1991). Furthermore, the 1-RMECC of 203.2 kg reported by Hollander et al. (2007), being slightly lower than the predicted 1-RMECC of 227.0 kg in the present study, likely reflects the difference in the cadence as well as differences in the strength between the groups of subjects used. Therefore, the predicted 1-RMECC being 164.8% of the 1-RM load may be considered a reasonable value.

The linear function was used to extrapolate the number of repetitions that would be performed to failure under loading conditions of 140, 150 and 160% 1-RM (Table 3). The load conditions from 110% through to 160% of 1-RM were then expressed relative to the predicted 1-RMECC of 164.8% (Table 3). These values are comparable to the repetition-load values presented by Baechle et al. (2008) for exercises utilizing the SSC. For example, Baechle et al. (2008) report 12, 8, 6, and 4 repetitions when using loads equivalent to 67, 80, 85 and 90% 1-RM. The values are strikingly similar to the 14.1, 9.3, 6.9 and 4.5 repetitions predicted in the present study when using loads equivalent to 66.7, 78.9, 85.0 and 91.0% 1-RMECC (or 110, 130, 140, and 150% 1-RM). It is therefore possible that researchers may start to use the repetition-load schemes presented here to investigate the kinematic and kinetic as well as metabolic responses to the eccentric-only bench press exercise using supra-maximal loads. For example, Ratamess et al. (2009) recommended that intermediate and advanced individuals perform resistance training exercises within a loading range from 1- to 12-RM, with an eventual emphasis on heavy loading (1- to 6-RM). For the eccentric-only bench, these loads would correspond to a range between 164.8% and 118.7% 1-RM (100% to 72% 1-RMECC), with loads between 164.8% and 143.9% 1- RM (100% to 87% 1-RMECC) employed for the heavier periods of the training program. These loads exceed those tested in the present study, which raises potential safety issues. Previous researchers had subjects perform an eccentric-only bench press with a load equivalent to 150% 1-RM (Murphy et al., 1994). However, these authors had subjects perform only a single repetition and they limited the range of motion to approximately 30o of elbow flexion to limit the potential for injury. Furthermore, the subjects performed the movement in a Smith machine, removing the requirement for spotters. Using the methodology of the present study, the requirement for spotters may preclude the use of loads greater than 130% 1-RM.

Caution may be required when using the repetition-load scheme developed in the present study. The accuracy of the scheme is predicated on the assumption that the relationship between the repetitions and load is linear. Although the scheme presented by Baechle et al. (2008) is also linear, there is some evidence that repetition-load schemes for the bench press performed utilizing the SSC may be curvilinear (LeSuer et al., 1997). It is possible that reflexive inhibition (Webber and Kriellaars, 1997) may alter the repetition load scheme proposed here. Furthermore, the scheme is likely to be accurate only for subjects possessing similar strength levels to those used in the present study and performing the exercise using free-weights as opposed to in a machine (Hoeger et al., 1990). However, the scheme presented provides an appropriate starting point to investigate the kinematic, kinetic and metabolic responses to eccentric-only bench press workouts, although the following issues need to be considered. Firstly, multiple sets of resistance exercises have been recommended in the development of factors such as muscular strength and hypertrophy (Ratamess et al., 2009), and it remains to be established how the repetition-load scheme is affected by the completion of multiple sets. Similarly, inter-set rest periods of 1–3 minutes have been recommended (Ratamess et al., 2009), but it is unclear if these periods are appropriate for eccentric-only contractions. There is evidence to suggest that eccentric contractions induce comparable fatigue to concentric contractions in the upper-body musculature (Mullaney and McHugh, 2006), although others report greater fatigue following eccentric contractions (Piitulainen et al., 2011). It is known that the physiological cost of eccentric contractions is less compared to other contraction types (Stauber, 1989). This has implications not only for the inter-set rest periods when using eccentric-only resistance exercises, but also for the potential strength and hypertrophy adaptations accrued following these exercises given the proposed importance of metabolic responses to resistance training exercises (Crewther et al., 2006). Finally, unaccustomed eccentric contractions are associated with tenderness and stiffness that develops 24 hours post-exercise known as delayed onset muscle soreness (DOMS) (Nosaka et al., 1991). The pain associated with DOMS appears to peak 1–3 days post-exercise, disappearing 7–10 days postexercise. Indeed, the occurrence of DOMS may preclude the use of eccentric-only exercises following the currently recommended frequency of 3–5 d·wk^−1^ (Ratamess et al., 2009), although the existence of the repeat-bout effect would be expected to rapidly protect against further muscle damage (McHugh, 2003). Clearly, further research is required before any recommendations can be made regarding the use of the eccentric-only bench press exercise in resistance training workouts.

The second purpose of the present study was to investigate the kinematic and kinetic differences between eccentric-only repetitions of the bench press performed to failure using supra-maximal loads of 110%, 120% and 130% of 1-RM. The use of a supra-maximal load equivalent to 130% 1-RM performed to failure produced significantly greater values for Total workmean as well as vertical Fmean, Workmean, and POmean compared to the repetitions performed with a load equivalent to 110% of 1-RM, despite a lower number of repetitions being completed at the same cadence. There were no differences in VL or Total workcum as a result of the different load conditions. Furthermore, the TUT did not differ between the different loading conditions tested meaning that the differences in power output were due to the amount of force exerted as opposed to the velocity of the barbell. The kinetic variables of force and work have been proposed to be important in the development of both strength and hypertrophy following a period of resistance training (Crewther et al., 2005). Therefore, from the current loads tested, 130% 1-RM would appear to provide a more appropriate stimulus than a load of 110% 1-RM, although these findings may be specific to the group of participants used in the present study and so future researchers should investigate if this proposal could be generalized.

## Conclusion

Given the proposed importance of eccentric muscle contractions in providing a more potent stimulus for both hypertrophy and strength gains following a period of resistance training (Roig et al., 2009; Schoenfeld, 2010), the repetition-load scheme presented here should provide a starting point for researchers to investigate the kinematic, kinetic and metabolic responses to eccentric-only bench press workouts. Ratamess et al. (2009) recommend a variety of repetition-load schemes for the long-term progression in muscular strength when using exercises utilizing the SSC, with schemes of 60–70% 1-RM performed for 8–12 repetitions progressing to loads equivalent to 80–100% 1-RM for 1–6 repetitions for an intermediate subject (an individual with approximately 6 months of consistent resistance training). Three sets with 3–5 minute rest periods performed at a frequency of 3–4 day·week^−1^ are deemed effective. Using the modified repetition-load scheme for the eccentric-only bench press, loads equivalent to 100–116% 1-RM would be performed initially, progressing to loads equivalent to 132–165% 1-RM, would be appropriate and the repetitions completed would be equivalent. For muscular hypertrophy, Ratamess et al. (2009) recommend loads of 70–85% 1-RM performed for 8–12 repetitions, with 3 sets interspersed with 1–2 minute rest periods performed 2–3 day·week^−1^. The corresponding loads for the eccentric-only bench would be 116–140% 1-RM, with equivalent repetitions expected to be completed. What remains to be determined is if the rest periods, sets, and frequency of workouts recommended by Ratamess et al. (2009) are appropriate for the modified repetition-load schemes applied to the eccentric-only bench press. Future researchers should investigate if the present findings could be generalized to larger groups of participants as well as establish the utility of the repetition-load scheme presented in our study.

## Figures and Tables

**Table 1:** The total number of repetitions, volume-load, and the mechanical variables of time under tension, mean total work, and cumulative total work recorded during eccentric-only bench press using supra-maximal loads of 110%, 120% and 130% of 1-RM performed to failure. Values are means ± standard deviations

Load condition	Total repetitions	VL	TUT_mean_ (s)	Total work_mean_ (J/kg^⅔^)	Total work_cum_ (J/kg^⅔^)
110% 1-RM	14.2 ± 3.5^*^	2132 ± 626	1.53 ± 0.42	0.25 ± 0.09^*^	3.57 ± 1.49
120% 1-RM	11.4 ± 5.1	1819 ± 664	1.59 ± 0.34	0.28 ± 0.11	3.37 ± 2.00
130% 1-RM	9.4 ± 3.8^*^	1629 ± 604	1.58 ± 0.31	0.30 ± 0.11^*^	2.98 ± 1.88

VL = volume-load; TUT_mean_ = mean time under tension calculated across all repetitions; Total work_mean_ = mean normalized work calculated across all repetitions; Total work_cum_ =cumulative normalized work calculated across all repetitions; 1-RM = 1-repetition maximum.

* Significant difference between 110% condition and 130% condition (p < 0.05).

**Table 2:** The mean values for the mechanical variables in the x, y, and z directions recorded during eccentric-only bench press using supra-maximal loads of 110%, 120% and 130% of 1-RM performed to failure. Values are means ± standard deviations

Load condition		F_mean_ (N/kg^⅔^)	Impulse_mean_ (Ns/kg^⅔^)	Work_mean_ (J/kg^⅔^)	PO_mean_ (W/kg^⅔^)
110% 1-RM	*x*	0.020 ± 0.008	0.023 ± 0.009	0.049 ± 0.037 × 10^2^	0.036 ± 0.028 × 10^2^
	*y*	0.072 ± 0.023	0.088 ± 0.040	0.004 ± 0.002	0.002 ± 0.001
	*z*	0.74 ± 0.22^*^	1.13 ± 0.64	0.24 ± 0.09^*^	0.17 ± 0.06^*^

120% 1-RM	*x*	0.017 ± 0.004	0.021 ± 0.009	0.038 ± 0.019 × 10^2^	0.023 ± 0.001 × 10^2^
	*y*	0.073 ± 0.026	0.095 ± 0.050	0.006 ± 0.004	0.003 ± 0.003
	*z*	0.76 ± 0.26	1.22 ± 0.73	0.28 ± 0.10	0.16 ± 0.05

130% 1-RM	*x*	0.019 ± 0.006	0.025 ± 0.009	0.041 ± 0.024 × 10^2^	0.028 ± 0.001 × 10^2^
	*y*	0.083 ± 0.032	0.106 ± 0.040	0.005 ± 0.004	0.003 ± 0.002
	*z*	0.85 ± 0.24^*^	1.31 ± 0.59	0.30 ± 0.10^*^	0.19 ± 0.06^*^

1-RM = 1-repetition maximum; x = mediolateral direction; y = anetrioposterior direction; z = vertical direction; F_mean_ = mean normalized average vertical force calculated across all repetitions; Impulse_mean_ = mean impulse of the vertical force calculated across all repetitions; PO_mean_ = mean normalized power output calculated across all repetitions; Work_mean_ = mean work calculated across all repetitions.

* Significant difference between 110% condition and 130% condition in the z direction (p < 0.05).

**Table 3:** The actual repetitions completed under the supra-maximal load conditions, the predicted number of repetitions completed, and the supra-maximal loading conditions expressed relative to the predicted eccentric-only one repetition maximum

Load condition	100% 1-RM	110% 1-RM	120% 1-RM	130% 1-RM	140% 1-RM	150% 1-RM	160% 1-RM
Actual repetitions	-	14.2	11.4	9.4	-	-	-
Predicted repetitions	16.5	14.1	11.7	9.3	6.9	4.5	2.1
Loading (%1-RM_ECC_)	60.7	66.7	72.8	78.9	85.0	91.0	97.1

*1-RM = 1-repetition maximum bench press utilizing the stretch-shortening cycle; Predicted repetitions = repetitions predicted from the equation y =* −*2.3889x + 16.481, where y = number of repetitions and x = loading (i.e. loading 0 = 100% 1-RM, 1 = 110% 1-RM, loading 2 = 120% 1-RM, etc.); Loading = the 1-RM loads expressed as a percentage of eccentric-only 1-RM predicted from the aforementioned equation; 1-RM_ECC_ = eccentric-only 1-repetition maximum (164.8% 1-RM).*
